# The first complete genomic sequences of African swine fever virus isolated in Poland

**DOI:** 10.1038/s41598-018-36823-0

**Published:** 2019-03-14

**Authors:** Natalia Mazur-Panasiuk, Grzegorz Woźniakowski, Krzysztof Niemczuk

**Affiliations:** 1 National Veterinary Research Institute (NVRI), Department of Swine Diseases, Partyzantów 57 Avenue, 24-100, Puławy, Poland; 2 National Veterinary Research Institute (NVRI), Director General, Partyzantów 57 Avenue, 24-100, Puławy, Poland

## Abstract

African swine fever (ASF) is a contagious, notifiable viral disease, which is considered a significant threat not only for European, but also for worldwide pork production, since recently the virus emerged within numerous Chinese pig herds. The disease was introduced in Poland in 2014 and up to the end of 2018,  213 outbreaks in pigs and 3347 cases in wild boars have been reported. The presented study describes the whole genome sequencing of seven Polish isolates, collected between 2016 and 2017, using next generation sequencing (NGS) technology. The complete, genomic sequences of these isolates were compared against five other closely related ASFV genomes, annotated in the NCBI database. The obtained sequences were from 189.393 to 189.405 bp long and encoded 187–190 open reading frames (ORFs). The isolates were grouped within genotype II and showed 99.941 to 99.956% nucleotide identity to the Georgia 2007/1 reference strain.

## Introduction

African swine fever, caused by the African swine fever virus (ASFV), is an OIE-notifiable, highly mortal disease affecting domestic pigs and wild boars. Currently neither therapy nor vaccine exist against the disease and the only applicable prevention methods rely on implementation of strict biosecurity measures, rapid laboratory diagnosis and stamping out the infected pig herds^1^. ASF was primary described in 1921 in Kenya^2^, but its recent re-emergence in 2007 in Georgia started its further spread to other Transcaucasian and Eastern-European countries including: Russia, Ukraine, Belarus, Lithuania, Latvia, Poland, Estonia, Moldova and more recently Czech Republic (June 2017), Romania (July 2017), Hungary (April 2018)^3–5^, Bulgaria (August 2018)^6^, and surprisingly also to Belgium (September 2018)^7^. Moreover, lately the disease has been introduced also into Chinese domestic pig population^8^, rising worldwide concerns about its further spread. In Poland ASF was firstly reported in February 2014^9^ and up to the end of September 2018, almost 2900 cases in wild boars and 213 outbreaks in pigs have been identified^10^. In spite of preventive measures taken within ASF-affected area, the number of affected animals is growing rapidly. Currently, ASFV spreads consistently towards the western part of Poland, at the end of 2017 the first cases in wild boars were confirmed close to Warsaw, another cluster of the disease emerged at the region close to the border with Kaliningrad Oblast (Russia)^10^ causing extreme concern in neigbouring western countries.

ASFV is a large, complex and multi-enveloped DNA virus, classified as the sole member of the genus *Asfivirus* within family *Asfarviridae*^11^. Its genome is composed of single, linear double stranded-DNA molecule, encoding genes essential in viral replication, virus assembly and egress as well as responsible for immunological interactions with the host^11^. The virus replicates predominantly in monocytes and macrophages, belonging to the mononuclear phagocyte system, although in the late stages of infection, other cell types may also be infected^12^. The swine monocyte/macrophage lineage is highly diverse and comprises a broad range of phenotypes at many maturation stages therefore showing different susceptibility to ASFV. Even thought, the pulmonary alveolar macrophages (PAMs) were suggested as more susceptible to infection in comparison to bone marrow or freshly-derived blood macrophages^13,14^. Since the field ASFV isolates do not replicate in conventional, continuous cell lines, for many years macrophages have been the only choice for isolation, propagation and titration of both field and adapted ASFV strains *in vitro*. In spite of many advantages of PAMs, the primary cells are difficult to obtain in sufficient amounts as required in numerous studies^15^. Additionally, the agreement of ethical commission is required. Moreover, some of the ASFV strains were adapted to grow in continuous cell lines, mostly derived from Green monkey, e.g. Vero, MS (stable monkey kidney cells, ECACC 91070510) or CV (ATCC® CCL-70™). This facilitated the development of more simple, repeatable, and quantitive method determining the ASFV infectivity, based on plaque formation^12,15^. However, this procedure was restricted only to culture adapted strains and could not be applied for field isolates. On the other hand, the continuous cell lines susceptible to ASFV infection, like IPAMs (immortalized pulmonary alveolar macrophages), COS-1 (monkey kidney fibroblasts) and WSL (wild boar lung macrophages), facilitated propagation both laboratory and field isolates^12,15^. In this report we describe attempts to isolate Polish field ASFV strains originating from wild boars and pigs in two types of cell cultures (IPAMs and PAMs). The isolated viruses were subjected for next generation sequencing (NGS) and whole genome analysis to reveal the genetic diversity of viruses currently circulating in Poland.

The genotyping approach on the basis of partial B646L gene sequencing encoding major ASFV capsid p72 protein and E183L gene encoding p54 protein, grouped Polish isolates within genotype II, and showed 100% nucleotide identity with all strains currently circulating in Europe^16^. The genetic analysis of the central variable region (CVR) within the B602L gene, showed the presence of unique amino acid tandem repeats, which were not revealed in strains circulating in the Caucasus region and Russia since 2007^17^. Additional studies on the intergenic region (IGR) sequence, spanning between ORFs encoding I73R and I329L genes, revealed the single insertion of a 10 nucleotide sequence (GGAATATATA), absent in strains obtained in Russia and Georgia. Nevertheless, the identical mutation was found in the sequences from Ukraine (Ukr12/Zapo), Belarus (Bel13/Grodno) and Lithuania (Lt14/1490), indicating molecular evolution among ASFV isolates circulating in Europe, and common ancestor of strains collected between 2013–2018 in countries westwards Russia^16^.

The study concerning molecular evolution of ASFV genes conducted by Frączyk *et al*.^18^ involving sequences obtained from cases and outbreaks reported in Poland between 2014–2015, revealed the genetic diversity within EP402R and MGF505-2R genes, indicating slow but consistent molecular evolution of these regions^18^. The results demonstrated that genetic variability of two analysed sequences is below 0.8% per analysed genomic region. Up to date, only a few whole genome sequences of European African swine fever isolates are available. Recently, Olesen *et al*.^19^ have conducted whole genome sequencing and a detailed analysis of results originated from Polish ASFV isolate ASFV/POL/2015/Podlaskie collected in 2015. The obtained sequence data (accession number: MH681419) showed 99.95% nucleotide identity with Georgia 2007/1 reference sequence, thus only 95 nt differs between compared sequences. In order to further our knowledge regarding the variability among strains currently circulating in Poland, and related European isolates, the whole genome sequences of 7 Polish isolates collected between 2016 and 2017 have been analysed. The results shed a light on the epidemiological studies concerning ASFV spread in Eurasia. Taking into consideration the unpredictability of human activity and limited dynamics of wild boar migration, the extensive analysis of NGS results may prove human influence in disease spread in Poland.

## Results

**Figure 1:**
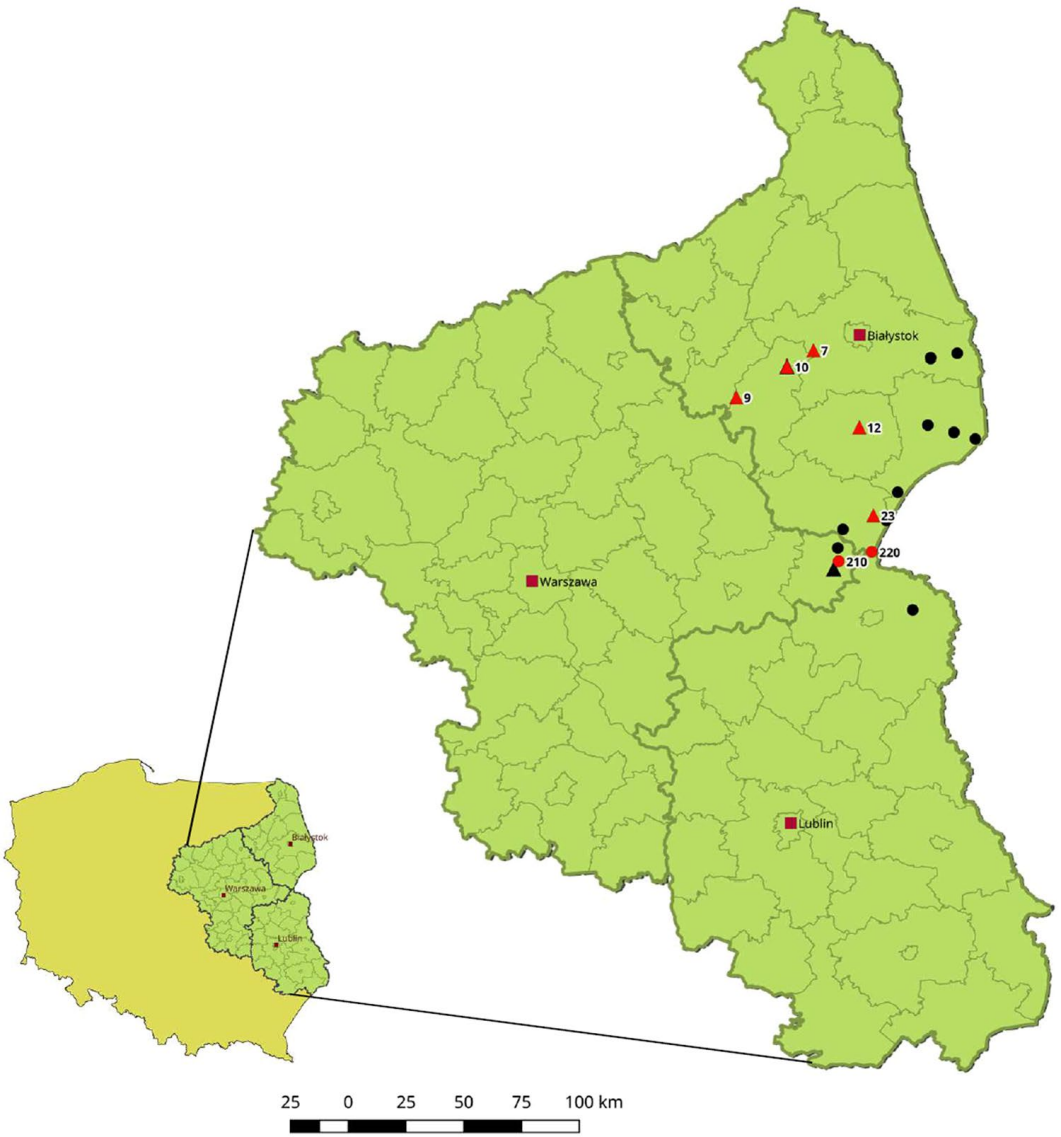
A map indicating geographical localization of investigated ASFV isolates. The numbers correspond to the whole genome sequenced isolates which are marked in red ( - Outbreaks,  - Cases). Other investigated samples are marked in black and are not numbered.

**Figure 2:**
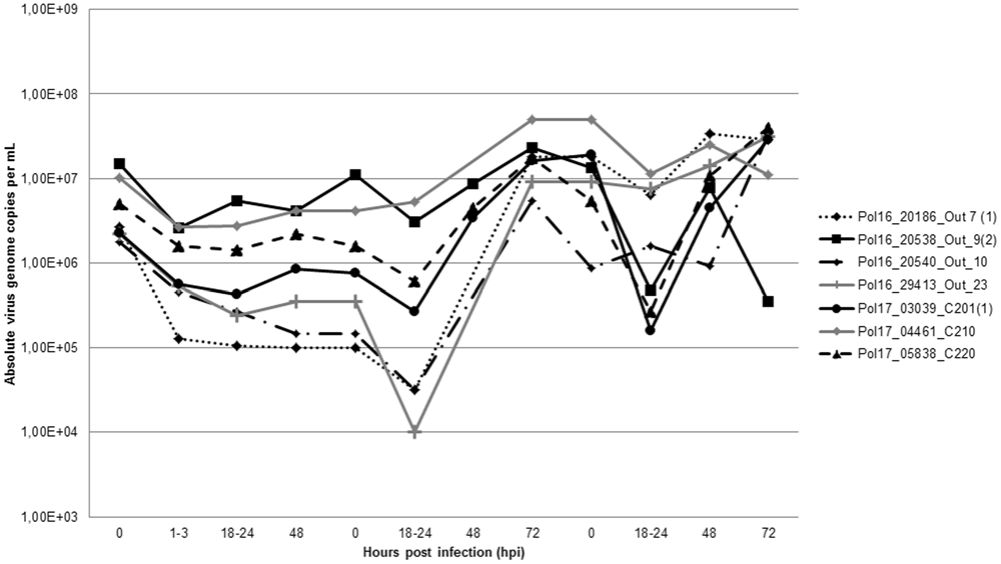
The replication dynamics of 7 investigated isolates during the infection (left part of chart), and two consecutive passages (central and right part of chart). The virus loads were expressed as absolute virus genome copies per milliliter.

**Figure 3:**
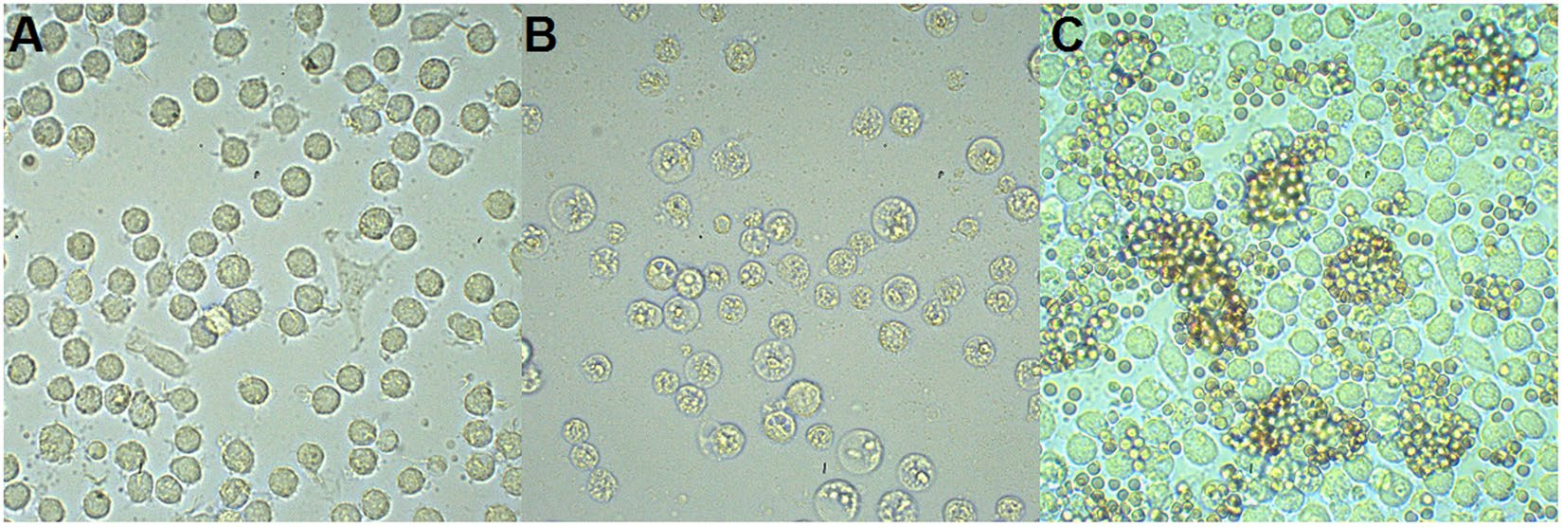
Infected PAM cells. (**A**) Mock-infected cells at 72 hpi, (**B**) Cytopathic effect observed at 72 hpi, (**C**) Hemadsorption at 48 hpi. Magnification 200X.

Infection of PAMs has been conducted using 48 virus suspensions originating from 14 domestic pigs (numbers of Outbreaks: 4, 7, 9, 10, 12, 20, 23) and 34 wild boars (numbers of Cases: 12, 14, 81, 91, 156, 160, 166, 167, 195, 201, 210, 211, 220, 221, 229, 232, 236, 235, 234, 245, 246, 249, 273) collected between 2014 and 2017. The detailed locations of the specimens are included in the Supplementary Table 2, as well in the Fig. 1. During the infection, the inoculum was removed at 1 to 3 hours postinfection (hpi), thus the quantity of absolute virus genome copies per milliliter (mL) decreased at 18 to 24 hpi in all samples. Nevertheless, 12 specimens at later stages of infection showed significant increase in this value, similar pattern was observed for these 12 samples in the subsequent passage (Fig. 2). The infected cells showed specific cytopathic effect (CPE), visible at 24 hpi. Infected macrophages were rounded, with massive vacuolization of the cytoplasm has been also observed. Some morphological differences between infected and uninfected cells are illustrated in Fig. 3A, B. To confirm ASFV replication and viable virus presence, the supernatants obtained in second passage were submitted to virus titration by hemadsorption assay (HAD), based on hemadsorption phenomenon unique to some ASFV genotypes, including investigated genotype II (Fig. 3C)^20^. All successfully propagated 12 strains yielded in titers between 6.32 and 7.32 log_10_ HAD_50_ ml^−1^ (50% hemadsorption doses per milliliter) (Supplementary Table 2).

The findings drawn on the basis of virus isolation in PAMs were consistent to real-time PCR results, which clearly showed, that viral replication was present in 12 specimens, of which 5 originated from wild boars and 7 originated from domestic pigs. The obtained 12 isolates were further used to infect IPAMs and passaged. Nevertheless, the whole virus load has been lost within the passage, suggesting IPAMs are not susceptible to Polish ASFV isolates (data not shown).

The IPAMs, as well as PAMs cells, were subjected to ASFV isolation in similar manner, using the same field-obtained inoculum. Nevertheless an established immortalized cell line did not show any observable cytopathic effect which may indicates ASFV replication. The results obtained by real-time PCR analysis of daily-collected samples from IPAMs showed that these cells do not support replication of the field Polish ASFV isolates.

Seven representatives were selected out of 12 isolates, namely originating from following cases: #201, #210, #220 and outbreaks: #7, #9, #10, #23, to subsequent whole genome sequencing. The NGS sequencing resulted in ASFV genome coverage from 20 to 40 times. The final assembled sequences ranged from 189.393 to 189.405 bp in length. The nucleotide genomic alignment was performed to compare Polish isolates with 10 fully annotated genomes (6544/OG10; Ba71V; Benin 97; E75; Georgia 2007/1; Ken05/Tk1; Ken06.Bus; L60; NHV; OURT 88/3) falling into various genotypes. The conducted analysis revealed from 80.57% to 99.95% identity with other annotated sequences. As expected, the Polish sequences showed the highest similarity to reference genotype II strain Georgia2007/1. The seven complete genomic sequences were deposited at NCBI GenBank accession numbers: MG939583-MG939589. The genomes contained from 187 to 190 ORFs and show an average 38.4% GC content.

**Figure 4:**
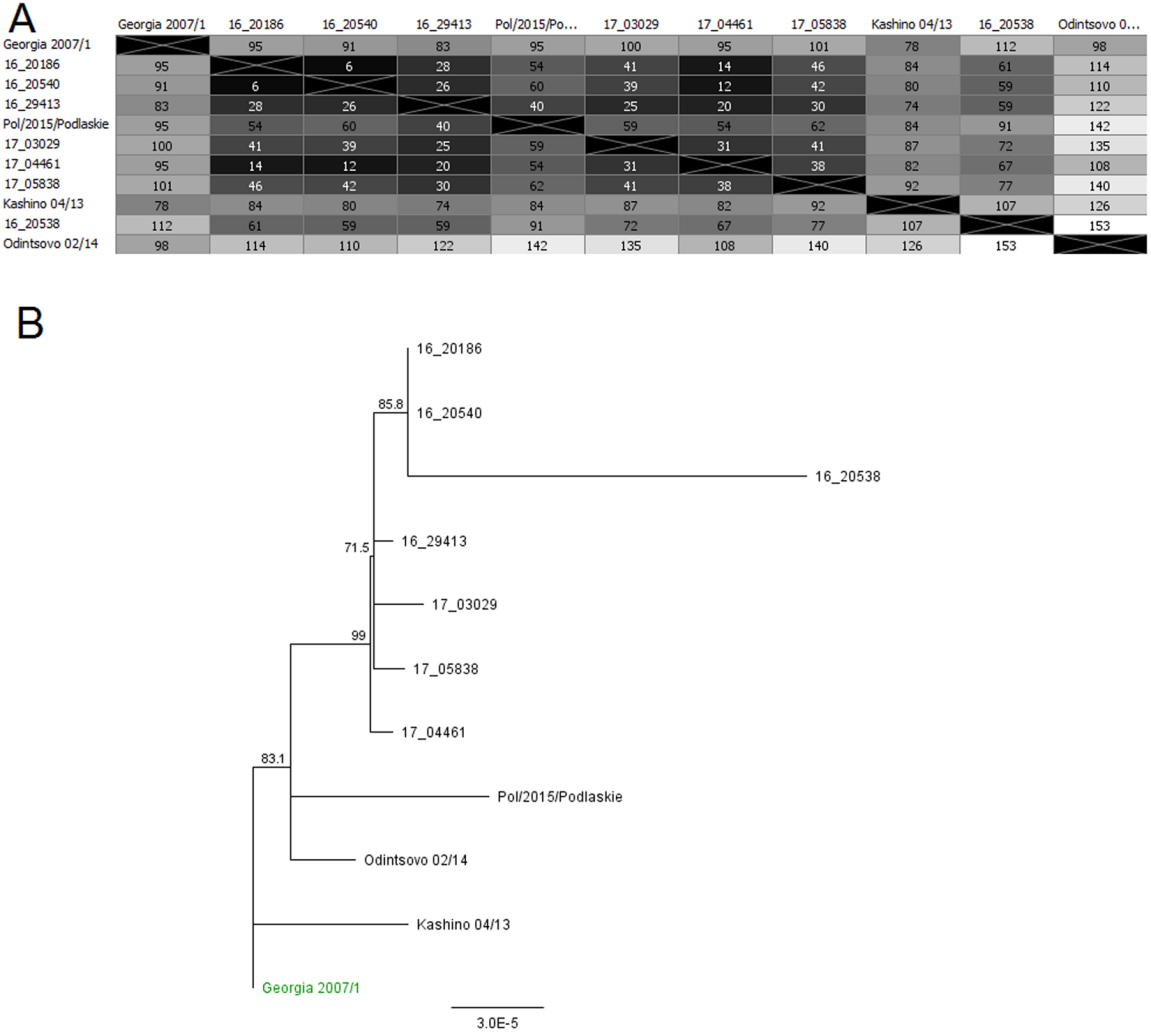
(**A**) Sequences identities matrix of the whole ASFV genomes. (**B**) Phylogram constructed on the basis of the aligned 7 genomic ASFV sequences and 4 reference sequences of Georgia 2007/1, Odintsovo 2/2014, Kashino 04/13 and ASFV/POL/2015/Podlaskie. The scale bar indicates the number of nucleotide substitutions per residue.

The conducted global alignment of 7 obtained ASFV genomes with the reference Georgia 2007/1^21^ (Accession number: FR682468.1), Odintsovo 02/2014^22^ (Accession number: KP843857.1), Kashino 04/13 (Accession number: KJ747406.1) and POL/2015/Podlaskie^19^ (Accession number: MH681419) genomes, showed minor variability which ranged from 99.919 (153 mismatches/differences) to 99.979% (40 mismatches/differences) (Fig. 4A). On the basis of the constructed phylogenetic tree it has been concluded that all examined Polish strains have the same genomic backbone with original Georgia 2007/1 strain (Fig. 4B). The BOOTSTRAP coefficient values were consistent and reached from 71.5 to 99.

The detailed comparative analyses of obtained genomes with Georgia 2007/1 revealed between 83 and 112 nt mismatches/differences, thus 99.941 to 99.956% similarity between Polish and reference Georgian strain. The variability between investigated sequences is summarized in the Supplementary Table 1. The alignment showed 55 variations within 35 genes in at least one Polish ASFV genomic sequence. Nevertheless only 26 differences were found in coding regions of all Polish isolates, moreover, 21 of them were previously detected in at least one of the aligned reference sequences analysed in this study (Supplementary Table 1). Remaining 5 variations were unique to Polish sequences; and refer to: MGF 110-7 L, MGF 505-5 R, K145R, I267L, DP60R genes. The first one was G-to-A transition, but the mutation was silent and did not induce any impact on the protein amino acid sequence. Other G-to-A transition was detected within MGF 505-5 R gene, and caused Val-to-Ile substitution. The C-to-A transversion within K145R gene causes another substitution, namely Ser-to-Tyr. The T-to-A transversion found within I267L gene, induce Ile-to-Phe substitution. The last variation unique to all Polish sequences was detected within DP60R gene, insertion of single A induces frame shift which leads to 14-nt elongation of open reading frame (ORF). The effects of these mutations on proteins functionality are the main issue of further investigations.

Except the described variations within the analysed encoding sequences present in all generated sequences of Polish ASFV isolates, additional 26 unique mutations might be detected within at least one (14%) of investigated seven sequences. Among the notable ones were: G-to-A transition observed within the MGF-505-4R ORF (Asp-to-Asn substitution in 3 out of 7 sequences, confirmed also in additional 3, unpublished whole genome sequences) and 14-nt insertion within ORF encoding O174L. The latter mutation was detected exclusively in 1 out of 7 sequences originated from the Case 201. Nevertheless, this tandem repeat was established also within additional 4 unpublished ASFV genomic sequences, originating from the Cases: 201 (second animal within the same case number), 211, 754 and the Outbreak 81. The mutation has been confirmed by conventional sequencing using a pair or primers designed to amplify 673-bp region of interest covering whole O174L gene.

Among the notable variations some mutations located within the QP383R, KP177R, CP204L, MGF 360-16 R ORFs and one insertion located at the intergenic region between MGF 300-1 L and MGF 300-4 L ORFs (position 21588–24589) were detected (Supplementary Table 1). It should be emphasized that all of them were reported previously by Olesen *et al*.^19^, therefore our analysis stands in line with this study. Two distinct indels (one deletion and one insertion of adenine) detected within QP383R ORF led to frame shift for only 12 consecutive codons. Interestingly, insertion of adenine within the KP177R ORF caused frame shift what resulted in truncation of putative protein (gene deletion). Nevertheless, second, in part compensatory ORF could be detected at the position starting 1nt downstream the insertion, which may result in folding of partial protein, 12 residues shortened at the N-terminus. Dual insertion of TT at CP204L ORF caused frame shift what resulted with premature stop codon and probable leads to truncation of last few residues at protein C-terminus. The frame shift caused by the insertion of A at MGF 360-16 R ORF caused fusion with the downstream ORF encoding DP63R. Insertion of A at position 21588–21589 referring to Georgia 2007/1, led to generate new ORF which encodes protein; showing 98.3% nucleotide, thus 97.5% amino-acid identity to MGF-300-2R encoded in other ASFV strains (eg. 6544/OG10; Ba71V; Benin 97; E75; L60; NHV; OURT 88/3).

The conducted analysis showed in total five non-synonymous mutations present in 100% of Polish sequences, resulting in single amino-acid substitution, namely: transitions within MGF 505-9 R (Lys-to-Glu) and NP419L (Asn-to-Ser), which were previously detected in other related genomes. The last three, namely: transitions within K145R (Ser-to-Tyr) and MGF 505-5 R (Val-to-Ile), and transversion within I267L ORF (Ile-to-Phe) ORFs, mentioned before, were detected exclusively within all investigated Polish isolates. The effect of these mutations on the proteins functionality is the subject matter for further investigation.

**Figure 5:**
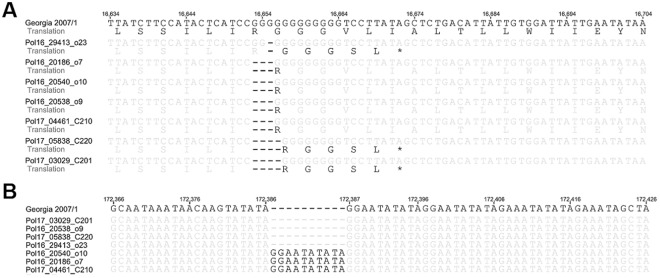
(**A**) Variations detected within ASFV_G_ACD_00290 gene resulting in probable truncation of putative protein (Outbreak: #23, Cases: #220, #201) or loss of one glycine codon (Outbreaks #7, #9, #10, Case #210). (**B**) Insertion of 10 additional nt (referring to the Georgia2007/1) within the intergenic region spanning between I73R and I329L ORFs. The variation was observed in 3 out of 7 Polish sequences.

Except the variations within coding regions, also a number of 66 mutations and single nucleotide polymorphisms (SNPs) were detected within non-coding regions, and 38 of them were found in all sequences generated in this study. In total 48 out of 66 variations were previously detected within at least one of aligned closely related genotype II isolates. Among the other important modifications within non-coding regions, was insertion of 10 nt (GGAATATATA) within the intergenic region spanning between I73R and I329R ORFs, detected only in 3 out of 7 sequences, referring to outbreaks 7^th^, 10^th^ and Case 210^th^ (Fig. 5B). 3 out of 7 isolates harbored the insertion of A within the ASFV G ACD 01760 ORF, which induced shift of frame leading to fusion with downstream ORF encoding I177L gene. Result of this mutation on protein function should be further investigated.

## Discussion

ASFV isolation in combination with hemadsorption assay is one of reference confirmatory tests in case of primary disease outbreak, although the second method is limited only to hemadsorbing strains^23,24^. Furthermore, virus isolation may not be possible due to the poor quality samples (particularly these originating from wild boars found dead). In this study, the samples subjected to viral isolation, were previously selected, and collected only from relatively well-preserved tissues, apart from these originated from bone marrow. Regarding the tested cell lines, only the PAMs were susceptible to infection with ASFV field isolates. Obviously, PAMs provide many advantages as they mimic natural infection, but its application has still some limitations associated with reproducibility, divergences between cells, laboriousness of collection and animal welfare issues^25^.

Our findings concerning IPAMs showed that this cells support replication only of following ASFV strains: Lisbon-61, Lillie SI/85^26^, Ba71V, ΔEP153R, E70, NHV^27^, Hinde (att.), Uganda (att.), CC83^12^. In the contrary, the results of the latest investigation regarding IPAMs susceptibility demonstrated, that this established cell line is not susceptible to infection neither with NHV/P68 nor Armenia/07^25^. The last-mentioned isolate was the only one genotype II strain tested on IPAMs, so our results stands in line with previous work which proved that IPAMs cell line does not support productive infection with this particular genotype, but the further analyses regarding Polish isolates infection in other established cell lines (e.g. WSL, COS-1) are required.

Seven generated complete genomic sequences of Polish ASFV isolates showed high level of similarity to other closely related European isolates annotated in GenBank database yet. The most divergent sequence was derived from strain Pol16_20538_O9 originating from outbreak #9 in Wysokie Mazowieckie poviat. This may indicate on the potential transmission of this virus from other – distanced area. However a minor variability on the level of 0.01–0.08 is not sufficient to draw such final conclusion. The minor variability has been observed as a point transitions within the 5′ and 3′ genomic region among other isolates as Pol16_29413_O23, 17_05838_C220 or Pol17_04461_C210 and Pol17_03029_C201. Interestingly, the isolate Pol16_29413_O23 originated from the outbreak #23 which occurred in 2016 in pig holding in Siemiatycze poviat. The strain seems to re-occur in 2017 as Pol17_05838_C220 strain from case #220 in the same poviat but also as Pol17_04461_C210 strain from case #210 and Pol17_03029_C201 from case #201 both from Łosice poviat. Other two strains namely Pol16_20540_O10 (outbreak #10 in Wysokie Mazowieckie poviat) and Pol16_20186_O7 (outbreak #7 in Bialystok poviat) were closely located at the phylogenetic tree. This may suggest the common source of the virus in these two pig holdings but the genetic diversity is not sufficient to prove it. The reference strains originating from Russia and Georgia formed separated branches suggesting their minor diversity from the sequence of Polish strains.

The aim of conducted study was to find the molecular relationship between sequences of ASF viruses currently circulating in Poland, which causes the yearly growing number of outbreaks and cases. Nevertheless, the diversity among the isolates proved to be insufficient to trace the origin of ASFV field isolates. Summarizing the mutations found in Polish sequences, included in the Supplementary Table 1, the final conclusion is that they differ from Georgian reference sequence. Interestingly, most of variations within the encoding sequences were previously detected in Russian or Polish whole genome ASFV sequences annotated in GenBank database. Moreover, Olesen *et al*., showed that some of detected indels were present also in Georgian isolate, indicating the possible existence of different variants of Georgia 2007/1 strain^19^. Nevertheless, it requires to be further investigated. Our study demonstrated that Polish strains have the common origin with Kashino04/13, Odintsovo 02/14 and ASFV/POL15/Podlaskie isolates, therefore might evolved and harbored numerous independent mutations, part of which could emerged after 2015. Nevertheless, this final conclusion requires further studies. The detected variations included mostly point insertions and deletions, some occurred within the ORFs encoding various proteins, which might be divided into three groups: uncharacterized (K145R, C84L, D129L, E184L, DP60R), functional (O174L, NP419L, QP383R), and structural proteins (KP177R, A151R, B602L, CP2475L, CP204L, E184L, E183L, E199L, I267L)^28^. Some mutations occurred within the ORFs encoding multigene families MGF505 and MGF360, which are closely linked with the ASFV virulence^29,30^, nevertheless, only five variations located within protein encoding sequences were unique to generated seven sequences, with the 100% frequency. Moreover, all of them have been confirmed in other, yet unpublished whole genome ASFV sequences, also collected between 2016 and 2017. Importantly, the 14-nt insertion detected within O174L gene in 1 out of 7 sequences, representing a tandem repeat, was confirmed by conventional sequencing of PCR products. Considering the minor variability of ASFV, it needs to be emphasized that such variation may present an excellent tool for subtyping of closely related viruses. Another considerable variability has been shown within intergenic region stretching between I73R and I329R, however our results stand in line with previous research evidencing the presence of this distinctive insertion in most of European Union ASFV strains, as well as in Belarussian, Ukrainian and some Russian strains^16^. This molecular marker is employed in standardized ASFV subtyping procedures.

The conducted study involved the ASFV whole genome sequences obtained from pig outbreaks, as well as from wild boar cases. No significant difference between sequences of various origin has been observed, nonetheless, few single nucleotide variations were detected. Among them is G-to-A transition within MGF-505-4R, which was found exclusively within outbreaks which emerged in August 2016 (Outbreaks: #7, #9, #10, and two additional sequences from Outbreaks #7 and #9, and also Outbreak #12). The similar pattern is observed within E199L gene, where C-to-T transition was detected within the analysed sequences. This may indicate common origin of all this outbreaks which are considered to be related with illegal pig trade, in particular due to very close onset dates (Supplementary Table 2). However, this minor similarity is not sufficient to draw such conclusion. Other unique variations were detected within E184L gene, namely three G-to-A transitions detected exclusively within the sequence originated from wild boar Case#201, however identical mutations were confirmed within partial sequence obtained from another animal within the same Case number, suggesting common origin of ASFV strains identified in this two dead wild boars.

The obtained results shed a light onto molecular epidemiology of currently circulating in Europe ASFV strains. We showed minor, but remarkable variability, proving slow, nevertheless still consistent evolution of viruses present in Poland. Determined whole genome sequences of highly virulent ASFV strains supplement current knowledge concerning virus spreading and evolution, as well as provide the backbone to such further investigations.

## Materials and methods

### Samples

In the study a panel of 48 ASFV-infected tissues samples, originating from the collection of the National Reference Laboratory for ASF at the National Veterinary Research Institute in Pulawy (Poland) was used. The specimens were comprised of various tissues of infected wild boars (found dead and hunted) and pigs. Small sections of ASF positive tissues were homogenized in sterile PBS buffer (10% wv^−1^), filtered (0.45 µm syringe filter, Sartorius) and applied to infect cell cultures for virus isolation and titer determination. Supplementary Table 2 features the detailed list of samples applied in the study.

### Cells

Pulmonary alveolar macrophages (PAMs) were obtained from Technical University of Denmark (DTU) and subcultured in RPMI 1640, 10% FBS, 1% Antibiotic-Antimycotic, 1x non-essential amino acids and 1 mM sodium pyruvate. All cultures were grown in 24-well microplate (10^6^ cells mL^−1^) at 37 °C in humidified atmosphere of air containing 5% CO_2_. After 18–24 h post-seeding the medium was removed and replaced with 300 µl of virus inoculum, followed by 2 h incubation at 37 °C to allow virus to adsorb. Subsequently the virus inoculum was removed, cells were washed twice with medium and incubated for 72 h. The 100 µl samples of medium were collected daily for further DNA extraction and real-time PCR analysis.

The immortalized pulmonary alveolar macrophages (IPAMs, 3D4/21) cell line used in this study was originally obtained from American Type Culture Collection (ATCC® CRL 2843™) and subcultured in RPMI 1640, supplemented with 10% FBS, 1x Antibiotic-Antimycotic, 10 mmol l^−1^ HEPES and 1.0 mmol l^−1^ sodium pyruvate. Sub-confluent cultures of IPAMs were infected accordingly to the above-mentioned manner, followed by daily 100 µl medium sample collection.

### Virus detection

The total DNA was extracted from collected medium using QIAamp DNA Mini Kit (Qiagen, Hilden, Germany) accordingly to the manufacturer’ recommendations. Thereafter, real-time PCR with an UPL probe was conducted as previously described^31^ using FastStart Universal Probe Master (Rox) kit (Roche, Mannheim, Germany) and UPL#162 probe (Roche). Quantification of ASFV genome was performed and calculated using the standard curve plotted on the basis of 10-fold dilution serious of standard VP-72 plasmid. The absolute quantity of ASFV genome was expressed as absolute virus genome copies per milliliter^32^.

To quantify the obtained virus strains, the hemadsorption assay (HAD) was performed as described elsewhere^20^. The titers were expressed as hemadsorbing doses per milliliter (HAD_50_ ml^−1^) calculated using Reed and Muench method^33^.

### Next generation sequencing

Seven representative isolates were selected out of all successfully isolated ASFV samples, and subjected to subsequent NGS sequencing. The obtained virus isolates were further propagated on PAMs, followed by collection of culture medium 5 days postinfection (dpi), which was used to purification of viral DNA. Briefly, cell supernatants were subjected to filtration through 0.22 μm syringe filter, subsequently precipitation buffer (20% Polyethylene glycol (PEG), 2.5 M sodium chloride) was added to obtain buffer-virus solution containing 8% PEG and agitated overnight at 4 °C. Virus containing protein fraction was pelleted by centrifugation at 18000 g for 90 min at 4 °C and resuspended in culture medium, then incubated with DNAse I (ThermoFisher Scientific), to digest contaminating cellular DNA. ASFV genomic DNA was extracted using High Pure PCR Template Preparation Kit, Roche. The next generation sequencing was conducted as an outsourcing service by Genomed, S.A. (Warsaw, Poland).

DNA libraries were prepared with NEBNext® UltraII DNA Library Prep Kit for Illumina (New England Biolabs, Ipswitch, USA), and sequenced on MiSeq instrument (Illumina) in PE250 mode. The number of obtained viral reads ranged from 22164 to 42630. The viral reads were filtered and trimmed using Cutadapt 1.9.1. software. The reads were mapped against the reference (accession number: FR682468.1) using CLC GenomicWorkbench. The seven complete genomic sequences of the following Polish ASFV strains: Pol16_20186_o7 (189401 bp), Pol16_20538_o9 (189399 bp), Pol16_20540_o10 (189405 bp), Pol16_29413_o23 (189393 bp), Pol17_03029_C201 (189405 bp), Pol17_04461_C210 (189401 bp), Pol17_05838_C220 (189393 bp) were obtained, with a mean coverage ranging from 19.98 to 40.8 reads per nt.

### PCR and conventional sequencing

In order to confirm duplication of 14-nt region within O174L gene, encoding polymerase beta-like protein^34^, a pair of primers amplifying 673-nt region of interest covering the mutation were designed. The primers binding sites were as follows: forward primer (5′- TGGCTCAGACGATATTTCAACTC-3′), binding sites: 128,160–128,182 and reverse primer (5′-GCCTCCACCACTTTGAACCAT-3′), binding sites: 128,813–128,832; with optimised annealing temperature as 46 °C, subsequently obtained PCR products were subjected to Sanger’s sequencing.

### Results analysis

The generated Polish ASFV genome sequences were aligned against four ASFV complete genomes available on NCBI (Accession numbers: FR682468.1; KP843857.1; MH681419; KJ747406.1) using the global alignment algorithm implemented inside Geneious software version R9 (Biomatters). The aligned sequences were subsequently mapped against the Georgia 2007/1 sequence in order to find variations, and the phylogenetic tree was build using Neighbor-Joining. A bootstrap analysis based on 1000 replicates was performed to assess the robustness of individual clades.

### Biosafety

National Veterinary Research Institute (NVRI) is licensed by the competent Polish authority to work with African swine fever virus. All experiment with infectious ASFV were performed in improved biosafety level 3 laboratories (BSL 3+).

## Electronic supplementary material

Supplementary information

## Data Availability

The sequences generated in this study were deposited at NCBI Genbank, accession numbers: MG939583 – MG939589. Other relevant data analysed during this study is included in this manuscript.

## References

[1] Sánchez-Vizcaíno JM, Mur L, Gomez-Villamandos JC, Carrasco L (2015). An Update on the Epidemiology and Pathology of African Swine Fever. J. Comp. Pathol..

[2] Montgomery ER, On A (1921). Form of Swine Fever Occurring in British EastAfrica (Kenya Colony). J. Comp. Pathol. Ther..

[3] EFSA AHAW Panel. African swine fever. *EFSA J*. **13**, 4163 (2015). 10.2903/j.efsa.2015.4163 PMC13151619 42109810

[4] Cortiñas Abrahantes, J. *et al*. Epidemiological analyses on African swine fever in the Baltic countries and Poland. *EFSA J*. **15**, (2017). 10.2903/j.efsa.2017.4732 PMC7010137 32625438

[5] World Organisation for Animal Health (OIE). African swine fever in Hungary. *Immediate notification ref OIE*: *26484*, *23/04/2018* (2018). Available at http://www.oie.int/wahis_2/public/wahid.php/Reviewreport/Review?page_refer=MapFullEventReport&reportid=26484 (Accessed 11^th^October 2018).

[6] World Organisation for Animal Health (OIE). African swine fever in Bulgaria. *Immediate notification ref OIE 27760* (2018). Available at: http://www.oie.int/wahis_2/public/wahid.php/Reviewreport/Review?page_refer=MapFullEventReport&reportid=27760 (Accessed 11^th^October 2018).

[7] World Organisation for Animal Health (OIE). African swine fever in Belgium. *Immediate notification ref OIE 27948* (2018). Available at http://www.oie.int/wahis_2/public/wahid.php/Reviewreport/Review?page_refer=MapFullEventReport&reportid=27948 (Accessed 11^th^October 2018).

[8] Zhou, X. *et al*. Emergence of African Swine Fever in China, 2018. *Transbound*. *Emerg*. *Dis*. 0–1 10.1111/tbed.12989 (2018). 10.1111/tbed.12989 30102848

[9] Pejsak, Z., Truszczyński, M., Niemczuk, K., Kozak, E. & Markowska-Daniel, I. Epidemiology of African Swine Fever in Poland since the detection of the first case. *Pol*. *J*. *Vet*. *Sci*. **17**, (2014). 10.2478/pjvs-2014-0097 25638980

[10] OIE World Animal Health Information System. Available at http://www.oie.int/wahis_2/public/wahid.php/Wahidhome/Home (Accessed 11^th^ October 2018) (2018).

[11] Dixon LK (2004). African swine fever virus proteins involved in evading host defence systems. Vet. Immunol. Immunopathol..

[12] de León P, Bustos MJ, Carrascosa AL (2013). Laboratory methods to study African swine fever virus. Virus Res..

[13] Rodriguez F (1996). African swine fever: morphopathology of a viral haemorrhagic disease. Vet. Rec..

[14] Blome S, Gabriel C, Beer M (2013). Pathogenesis of African swine fever in domestic pigs and European wild boar. Virus Res..

[15] Hurtado C, Bustos MJ, Carrascosa AL (2010). The use of COS-1 cells for studies of field and laboratory African swine fever virus samples. J. Virol. Methods.

[16] Gallardo C (2014). Genetic Variation among African Swine Fever Genotype II Viruses, Eastern and Central Europe. Emerg. Infect. Dis..

[17] Malogolovkin A, Yelsukova A, Gallardo C, Tsybanov S, Kolbasov D (2012). Molecular characterization of African swine fever virus isolates originating from outbreaks in the Russian Federation between 2007 and 2011. Vet. Microbiol..

[18] Frączyk M (2016). Evolution of African swine fever virus genes related to evasion of host immune response. Vet. Microbiol..

[19] Olesen AS (2018). Complete genome sequence of an African swine fever virus (ASFV POL/2015/Podlaskie) determined directly from pig erythrocyte-associated nucleic acid. J. Virol. Methods.

[20] Malmquist WA, Hay D (1960). Hemadsorption and cytopathic effect produced by African Swine Fever virus in swine bone marrow and buffy coat cultures. Am. J. Vet. Res..

[21] Chapman DAG (2011). Genomic analysis of highly virulent Georgia 2007/1 isolate of African swine fever virus. Emerg. Infect. Dis..

[22] Elsukova A, Shevchenko I, Varentsova A, Puzankova O (2017). Biological properties of African swine fever virus Odintsovo 02/14 isolate and its genome analysis. Int J Environ. Agric Res..

[23] Gallardo C (2015). Assessment of African Swine Fever Diagnostic Techniques as a Response to the Epidemic Outbreaks in Eastern European Union Countries: How To Improve Surveillance and Control Programs. J. Clin. Microbiol..

[24] Sánchez-Vizcaíno JM, Mur L, Gomez-Villamandos JC, Carrasco L (2015). An Update on the Epidemiology and Pathology of African Swine Fever. J. Comp. Pathol..

[25] Sánchez EG (2017). Phenotyping and susceptibility of established porcine cells lines to African Swine Fever Virus infection and viral production. Sci. Rep..

[26] Weingartl H, Sabara M, Pasick J, van Moorlehem E, Babiuk L (2002). Continuous porcine cell lines developed from alveolar macrophages. J. Virol. Methods.

[27] Carrascosa, A. L., Bustos, M. J. & de Leon, P. Methods for growing and titrating african swine fever virus: Field and laboratory samples. *Curr*. *Protoc*. *Cell Biol*. 1–25, 10.1002/0471143030.cb2614s53 (2011). 10.1002/0471143030.cb2614s53 22161547

[28] Dixon LK, Chapman DAG, Netherton CL, Upton C (2013). African swine fever virus replication and genomics. Virus Res..

[29] O’Donnell V (2015). African Swine Fever Virus Georgia Isolate Harboring Deletions of MGF360 and MGF505 Genes Is Attenuated in Swine and Confers Protection against Challenge with Virulent Parental Virus. J. Virol..

[30] Krug PW (2015). The Progressive Adaptation of a Georgian Isolate of African Swine Fever Virus to Vero Cells Leads to a Gradual Attenuation of Virulence in Swine Corresponding to Major Modifications of the Viral Genome. J. Virol..

[31] Fernández-Pinero J (2013). Molecular Diagnosis of African Swine Fever by a New Real-Time PCR Using Universal Probe Library. Transbound. Emerg. Dis..

[32] King DP (2003). Development of a TaqMan® PCR assay with internal amplification control for the detection of African swine fever virus. J. Virol. Methods.

[33] Reed LJ, Muench H (1938). A simple method of estimating fifty per cent endpoints. Am. J. Epidemiol..

[34] Redrejo-Rodriguez M, Rodriguez JM, Suarez C, Salas J, Salas ML (2013). Involvement of the Reparative DNA Polymerase Pol X of African Swine Fever Virus in the Maintenance of Viral Genome Stability *In Vivo*. J. Virol..

