# Supplementary material for: The first complete genomic sequences of African swine fever virus isolated in Poland
Source: Sci Rep. 2019 Mar 14;9:4556. doi: 10.1038/s41598-018-36823-0 (PMC6418159; doi:10.1038/s41598-018-36823-0)
Supplement: Supplementary file 1 — Supplementary information [file 41598_2018_36823_MOESM1_ESM.pdf]

The first complete genomic sequences of African swine fever virus isolated in Poland.

Natalia Mazur-Panasiuk[1*], Grzegorz Woźniakowski[1] and Krzysztof Niemczuk[2]

[1]*National Veterinary Research Institute (NVRI), Department of Swine Diseases, Partyzantów 57 Avenue, 24-100 Puławy, Poland*

[2]*National Veterinary Research Institute (NVRI), Director General, Partyzantów 57 Avenue, 24-100 Pulawy, Poland*

[*]Corresponding author. Tel. +48818893062

Email address: natalia.mazur@piwet.pulawy.pl

Supplementary table 1. Summary of genetic variations detected between seven Polish ASFV sequences and the reference strain, Georgia 2007/1, and their probable implications on the protein sequence. The occurrence of detected variations was indicated also in previously sequenced closely related whole ASFV genomes.

[a] Positions are referring to reference, Georgia 2007/1
[b] The frequency of variation is referring to the seven Polish isolates.

	Polymorphism Type	Left flanking position[a]	Right flanking position[a]	Nucleotide change	CDS	Amino Acid Change	Effect on protein sequence	Frequency[b]	Occurrence				Comment:
									This study	Kashino 04/13	Odintsovo 02/14	Pol/2015/ Podlaskie	
1.	Indel	412	413	insertion A	terminal inverted repeat			100.0%		Yes	No	Yes	
2.	Indel	432	433	insertion C				71.4%	Pol16_20186_o7 Pol16_20538_o9 Pol16_20540_o10 Pol16_29413_o23 Pol17_03029_C201 Pol17_04461_C210, Pol17_05838_C220	Yes	Yes	Yes	
3.	Indel	433	434	insertion T				100.0%		Yes	Yes	Yes	
4.	Indel	440	441	insertion A				100.0%		Yes	Yes	Yes	
5.	Indel	1604	1607	deletion TT	MGF 360-1L		Frame shift	100.0%		Yes	Yes	Yes	
6.	Indel	1622	1623	insertion T	MGF 360-1L		Frame shift	100.0%		Yes	Yes	Yes	
7.	Indel	3246	3247	insertion A	KP177R		Frame shift	100.0%		No	Yes	No	37 nt shortened ORF, impact on protein N-terminus
8.	SNP (transistion)	3328	3330	T -> C	KP177R		Frame shift	14%	Pol16_20538_o9	No	No	No	
9.	SNP	3331	3333	T -> C	KP177R		Frame shift	14%	Pol16_20538_o9	No	No	No	
10.	Indel	3333	3334	insertion G	KP177R		Frame shift	14%	Pol16_20538_o9	No	No	No	175 nt shortened ORF in comparison to other Polish sequences,

	Polymorphism Type	Left flanking position[a]	Right flanking position[a]	Nucleotide change	CDS	Amino Acid Change	Effect on protein sequence	Frequency[b]	Occurrence				Comment:
									This study	Kashino 04/13	Odintsovo 02/14	Pol/2015/ Podlaskie	
													impact on protein N-terminus
11.	Indel	3861	3862	insertion A				100.0%		Yes	No	Yes	
12.	Indel	5670	5671	insertion A	ASFV G ACD 00070		Frame shift	100.0%		Yes	Yes	Yes	46 nt shortened ORF
13.	Indel	5869	5870	insertion A				100.0%		No	Yes	Yes	
14.	SNP (transition)	6094	6096	C -> T	MGF 110-1L		Truncation	100.0%		No	Yes	No	54 nt shortened ORF,
15.	Indel	7823	7824	insertion A	ASFV G ACD 00120		Frame shift	100.0%		Yes	No	Yes	Impact on protein N-terminus
16.	Indel	7940	7941	insertion A	ASFV G ACD 00120		Frame shift	100.0%		Yes	Yes	Yes	Impact on protein N-terminus
17.	Indel	8505	8506	insertion A				100.0%		Yes	Yes	Yes	
18.	SNP (transition)	9700	9702	G -> A	MGF 110-7L		None	100.0%		No	No	No	Confirmed within other 7 unpublished whole genome sequences
19.	Indel	10465	10466	insertion A				100.0%		Yes	No	Yes	
20.	Indel	11609	11610	insertion A	ASFV G ACD 00190		Frame shift	85.7%	Pol16_20186_o7 Pol16_20540_o10 Pol16_29413_o23 Pol17_03029_C201 Pol17_04461_C210 Pol17_05838_C220	No	No	No	19 nt shortened ORF Confirmed within other 4 unpublished whole genome sequences
21.	Indel	13265	13269	Deletion of 2xC's to 5xC's	MGF-110-14L		Frame shift	57%	Pol17_04461_C210 Pol16_29413_o23 Pol17_03029_C201 Pol17_05838_C220	No	No	No	5xC: 20 nt elongated ORF 4xC: Fusion with downstream ORF (MGF-110-11L) 3xC: 3 nt shortened ORF Confirmed within other 3

	Polymorphism Type	Left flanking position[a]	Right flanking position[a]	Nucleotide change	CDS	Amino Acid Change	Effect on protein sequence	Frequency[b]	Occurrence				Comment:
									This study	Kashino 04/13	Odintsovo 02/14	Pol/2015/ Podlaskie	
													unpublished whole genome seuences
22.	Indel	13665	13666	insertion A	ASFV G ACD 00240		Frame shift	100.0%		Yes	No	Yes	26 nt elongated ORF
23.	SNP (transition)	14695	14697	A -> C	MGF-110-13L		None	14%	Pol16_20538_o9	No	No	No	Confirmed within other 2 unpublished whole genome sequence
24.		14713	14717	ATGTT -> CCCCC	MGF-110-13L	WT -> GG	Substitution	14%	Pol16_20538_o9	No	No	No	Confirmed within other 3 unpublished whole genome sequence
25.	Indel	16661	16664	deletion of 1xG to 4xG's	ASFV G ACD 00290		Frame shift/deletion of G codon	100%	Pol16_29413_o23	Yes* *GG deletion within 16662-16663	Yes* *GG deletion within 16662-16663	No	Impact on protein N-terminus/premature Stop codon
26.	Indel	16878	16880	deletion G				71%	Pol16_20186_o7 Pol16_20538_o9 Pol16_20540_o10 Pol16_29413_o23 Pol17_03029_C201 Pol17_04461_C210	Yes	Yes	Yes* *deletion of GG at position 16877-16878	
27.	Indel	18736	18737	insertion T				100.0%		Yes	No	Yes	
28.	Indel	19034	19039	deletion GGGG	ASFV_G_ACD_00350		Truncation	14%	Pol16_20186_o7	No	No	Yes * *GGGGGG deletion within 19033-19038	83 nt shortened ORF
29.	SNP (transition)	19038	19040	T -> A	ASFV_G_ACD_00351	S -> T	Substitution	14%	Pol17_04461_C210	No	No	No	
30.	Indel	19461	19462	insertion T				85.7%	Pol16_20186_o7 Pol16_20540_o10 Pol16_29413_o23 Pol17_03029_C201 Pol17_04461_C210 Pol17_05838_C220	No	No	Yes	
31.	SNP (transition)	19477	19479	C -> T				14%	Pol16_20538_o9	No	No	No	

	Polymorphism Type	Left flanking position[a]	Right flanking position[a]	Nucleotide change	CDS	Amino Acid Change	Effect on protein sequence	Frequency[b]	Occurrence				Comment:
									This study	Kashino 04/13	Odintsovo 02/14	Pol/2015/ Podlaskie	
32.	Indel	20669	20670	insertion A				100.0%		Yes	Yes	Yes	
33.	Indel	20833	20836	deletion GG				14%	Pol16_29413_o23	No	No	Yes* *G deletion within 20834-10836	
34.	Indel	20835	20836	insertion G				14%	Pol16_20540_o10	Yes	Yes	No	
35.	Indel	21588	21589	insertion A	new orf - MGF-300-2R			100.0%		Yes	No	Yes	New ORF developed
36.	Indel	23027	23028	insertion A				57.1%	Pol16_20540_o10 Pol16_29413_o23 Pol17_03029_C201 Pol17_04461_C210 Pol17_05838_C220	Yes	No	Yes	
37.	Indel	26455	26456	insertion T				85.7%	Pol16_20186_o7 Pol16_20540_o10 Pol16_29413_o23 Pol17_03029_C201 Pol17_04461_C210 Pol17_05838_C220	No	No	Yes	
38.	Indel	31678	31679	insertion A				100.0%		Yes	No	Yes	
39.	SNP (transition)	36068	36070	G -> A	MGF-505-4R	D -> N	Substitution	43%	Pol16_20186_o7 Pol16_20538_o9 Pol16_20540_o10	No	No	No	Confirmed within other 3 unpublished whole genome sequences
40.	Indel	37326	37327	insertion A				14%	Pol16_20538_o9	No	No	No	
41.	SNP (transition)	38331	38333	G -> A	MGF 505-5R	V -> I	Substitution	100.0%		No	No	No	Confirmed within other 7 unpublished whole genome sequences
42.	Indel	39024	39025	insertion T				100.0%		No	No	Yes* *TT insertion	

	Polymorphism Type	Left flanking position[a]	Right flanking position[a]	Nucleotide change	CDS	Amino Acid Change	Effect on protein sequence	Frequency[b]	Occurrence				Comment:
									This study	Kashino 04/13	Odintsovo 02/14	Pol/2015/ Podlaskie	
43.	Indel	39795	39796	insertion G	MGF-505-6R		Frame shift	14%	Pol17_05838_C220	No	No	No	787 nt shortened ORF, second compensatory ORF at position 39777-40618
44.	Indel	40730	40731	insertion T				100.0%		No	No	Yes	
45.	Indel	42616	42617	insertion T				100.0%		No	No	Yes	
46.	SNP (transition)	43598	43600	A -> G	MGF 505-9R	K -> E	Substitution	100.0%		No	Yes	No	
47.	Indel	44547	44548	insertion T				100.0%		Yes	No	Yes	
48.	Indel	47321	47322	insertion A				100.0%		Yes	Yes	Yes	
49.	Indel	48 663	48 664	insertion A	A151R		Frame shift	100.0%		No	No	Yes	22 nt shortened ORF
50.	Indel	49220	49221	insertion A				14%	Pol16_20538_o9	No	No	No	
51.	Indel	49225	49226	insertion T				14%	Pol16_20538_o9	No	No	No	
52.	SNP (transition)	49230	49232	T -> G				14%	Pol16_20538_o9	No	No	No	
53.	Indel	49235	49236	insertion A				14%	Pol16_20538_o9	No	No	No	
54.	SNP (transition)	49240	49242	A -> C				14%	Pol16_20538_o9	No	No	No	
55.	Indel	49250	49251	insertion C				14%	Pol16_20538_o9	No	No	No	
56.	Indel	55974	55975	insertion A				85.7%	Pol16_20186_o7 Pol16_20540_o10 Pol16_29413_o23	Yes	No	Yes	

	Polymorphism Type	Left flanking position[a]	Right flanking position[a]	Nucleotide change	CDS	Amino Acid Change	Effect on protein sequence	Frequency[b]	Occurrence				Comment:
									This study	Kashino 04/13	Odintsovo 02/14	Pol/2015/ Podlaskie	
									Pol17_03029_C201<br>Pol17_04461_C210<br>Pol17_05838_C220				
57.	Indel	63845	63846	insertion T				100.0%		No	Yes	Yes	
58.	Indel	63860	63861	insertion A				100.0%		No	Yes	Yes	
59.	Indel	63867	63868	insertion T				100.0%		No	Yes	Yes	
60.	Indel	63876	63877	insertion T				100.0%		No	No	Yes	
61.	SNP (transversion)	65166	65168	C -> A	K145R	S -> Y	Substitution	100.0%		No	No	No	Confirmed within other 6 unpublished whole genome sequences
62.	Indel	81525	81526	insertion T	C84L		Frame shift	100.0%		No	No	Yes	10 nt shortened ORF
63.	Indel	81619	81620	insertion A				85.7%	Pol16_20186_o7<br>Pol16_20540_o10<br>Pol16_29413_o23<br>Pol17_03029_C201<br>Pol17_04461_C210<br>Pol17_05838_C220	Yes	No	Yes	
64.	SNP (transistion)	101037	101039	C -> T	B602L	G->D	Substitution	14%	Pol16_20538_o9	No	No	No	
65.	SNP (transistion)	101044	101046	C -> T	B602L	E->K	Substitution	14%	Pol16_20538_o9	No	No	No	
66.	SNP (transistion)	101047	101049	G -> T	B602L	Q->K	Substitution	14%	Pol16_20538_o9	No	No	No	
67.	SNP (transversion)	101049	101051	A -> T	B602L	I->K	Substitution	14%	Pol16_20538_o9	No	No	No	
68.	Indel	113166	113167	insertion A				100.0%		Yes	Yes	Yes	

	Polymorphism Type	Left flanking position[a]	Right flanking position[a]	Nucleotide change	CDS	Amino Acid Change	Effect on protein sequence	Frequency[b]	Occurrence				Comment:
									This study	Kashino 04/13	Odintsovo 02/14	Pol/2015/ Podlaskie	
69.	Indel	117181	117182	insertion T				85.7%	Pol16_20186_o7 Pol16_20540_o10 Pol16_29413_o23 Pol17_03029_C201 Pol17_04461_C210 Pol17_05838_C220	No	No	Yes	
70.	SNP (transition)	117900	117902	G -> T	CP2475L	Q->K	Substitution	14%	Pol17_05838_C220	No	No	No	
71.	SNP (transistion)	117931	117933	C -> A	CP2475L		None	14%	Pol17_05838_C220	No	No	No	
72.	SNP (transversion)	118859	118861	A -> T	CP2475L		None	14%	Pol16_20538_o9	No	No	No	Confirmed within other 1 unpublished whole genome sequences
73.	Indel	118878	118879	insertion A	CP2475L		Frame shift	14%	Pol16_20538_o9	No	No	No	1594 nt shortened ORF Confirmed within other 1 unpublished whole genome sequences
74.	Indel	124704	124705	insertion A				100.0%		No	No	Yes	
75.	Indel	124803	124804	Insertion TT	CP204L		Frame shift	100.0%		Yes* *T insertion	Yes* *T insertion Yes	Yes	23 nt shortened ORF
76.	Indel	128301	128302	insertion CAGTAGT GATTTTT	o174L		Frame shift	14%	Pol17_03029_C201	No	No	No	35 nt shortened ORF, tandem repeat Confirmed within other 4 unpublished whole genome sequences Confirmed by conventional sequencing
77.	Indel	133463	133464	insertion A				100.0%		Yes	No	Yes	
78.	SNP (transition)	133520	133522	T -> C	NP419L	N -> S	Substitution	100.0%		No	Yes	No	
79.	Indel	138421	138422	insertion T	D129L		Frame shift	100.0%		No	No	Yes	
80.	Indel	143757	143758	insertion A				100.0%		Yes	No	Yes	

	Polymorphism Type	Left flanking position[a]	Right flanking position[a]	Nucleotide change	CDS	Amino Acid Change	Effect on protein sequence	Frequency[b]	Occurrence				Comment:
									This study	Kashino 04/13	Odintsovo 02/14	Pol/2015/ Podlaskie	
81.	Indel	152774	152775	insertion C				14%	Pol16_20538_o9	No	No	No	Confirmed within other 1 unpublished whole genome sequences
82.	Indel	152777	152780	insertion AC				14%	Pol16_20538_o9	No	No	No	Confirmed within other 1 unpublished whole genome sequences
83.	Indel	152786	152789	deletion CA				14%	Pol16_20538_o9	No	No	No	Confirmed within other 2 unpublished whole genome sequences
84.	Indel	152792	152793	insertion A				100.0%		No	No	Yes	
85.	Indel	161251	161 253	deletion A	QP383R		Frame shift	100.0%		Yes	Yes	Yes	Frame shift for 12 consecutive codons
86.	Indel	161292	161293	insertion A	QP383R		Frame shift	100.0%		Yes	Yes	Yes	Return to initial ORF
87.	SNP (transition)	161824	161826	C->T	E184L	M->I	Substitution	14%	Pol16_29413_o23	No	No	No	
88.	SNP (transition)	162597	162599	G -> A	E183L	A->V	Substitution	14%	Pol17_03029_C201	No	No	No	Confirmed within other 1 unpublished whole genome sequence
89.	SNP (transition)	162634	162636	G -> A	E183L		None	14%	Pol17_03029_C201	No	No	No	Confirmed within other 1 unpublished whole genome sequence
90.	SNP (transition)	162698	162700	G-> A	E183L		None	14%	Pol17_03029_C201	No	No	No	Confirmed within other 1 unpublished whole genome sequence
91.	SNP (transition)	166071	166073	C-> T	E199L	E->K	Substitution	43%	Pol16_20186_o7 Pol16_20538_o9 Pol16_20540_o10	No	No	No	Confirmed within other 3 unpublished whole genome sequences
92.	Indel	167763	167764	insertion A				100.0%		No	No	Yes	
93.	Indel	169454	169455	insertion T				100.0%		Yes	No	Yes	
94.	Indel	169462	169463	insertion A				100%		Yes	Yes* *T insertion	Yes	

	Polymorphism Type	Left flanking position[a]	Right flanking position[a]	Nucleotide change	CDS	Amino Acid Change	Effect on protein sequence	Frequency[b]	Occurrence				Comment:
									This study	Kashino 04/13	Odintsovo 02/14	Pol/2015/ Podlaskie	
95.	Indel	169741	169742	insertion T	I267L		Frame shift	100.0%		Yes	No	Yes	37 nt shortened ORF
96.	SNP (transversion)	169861	169863	T -> A	I267L	I -> F	Substitution	100.0%		No	No	No	Confirmed within other 6 unpublished whole genome sequences
97.	Indel	172017	172018	insertion T				100.0%		No	No	Yes	
98.	Indel	172406	172407	insertion GGAATAT ATA	Intergenic region I73R-I329R			43%	Pol16_20186_o7<br>Pol16_20540_o10<br>Pol17_04461_C210	No	Yes	No	
99.	Indel	173565	173566	insertion A				100.0%		Yes	Yes	Yes	
100.	Indel	174954	174955	insertion A	ASFV G ACD 01760		Frame shift	85.7%	Pol16_20186_o7<br>Pol16_20540_o10<br>Pol16_29413_o23<br>Pol17_03029_C201<br>Pol17_04461_C210<br>Pol17_05838_C220	Yes	No	Yes	Fusion with downstream ORF (I77L)
101.	Indel	175632	175633	Insertion A				100.0%		Yes	No	Yes	
102.	SNP (transition)	177473	177475	G -> A	MGF 360-16R		None	57.1%	Pol16_20186_o7<br>Pol16_20538_o9<br>Pol16_20540_o10<br>Pol16_29413_o23	No	No	No	
103.	Indel	177493	177494	Insertion A	MGF 360-16R		Frame shift	100.0%		Yes	No	Yes	Fusion with downstream ORF (DP63R)
104.	Indel	179379	179380	insertion A				100.0%		No	No	Yes	
105.	Indel	183305	183306	insertion T				100.0%		Yes	Yes	Yes	
106.	Indel	183314	183315	insertion T				100.0%		Yes	No	Yes	
107.	Indel	184967	184969	deletion T	ASFV G ACD 01940		Frame shift	100.0%		Yes	Yes	Yes	28 nt elongated ORF

	Polymorphism Type	Left flanking position[a]	Right flanking position[a]	Nucleotide change	CDS	Amino Acid Change	Effect on protein sequence	Frequency[b]	Occurrence				Comment:
									This study	Kashino 04/13	Odintsovo 02/14	Pol/2015/ Podlaskie	
108.	Indel	186352	186353	insertion T				100.0%		Yes	No	Yes	
109.	Indel	189108	189109	insertion A	DP60R		Frame shift	100.0%		No	No	No	14 nt elongated ORF Confirmed within other 8 unpublished whole genome sequences
110.		189183	189186	CT -> AA				14%	Pol16_20538_o9	No	No	No	
111.	SNP (transistion)	189186	189188	G-> T				14%	Pol16_20538_o9	No	No	No	
112.	SNP (transistion)	189188	189190	G -> T				14%	Pol16_20538_o9	No	No	No	
113.	Indel	189194	189195	insertion TTT				14%	Pol16_20538_o9	No	No	No	
114.	SNP (transversion)	189218	189220	C -> G				14%	Pol16_20538_o9	No	No	No	
115.	SNP (transistion)	189222	189224	G ->A				100%		No	No	Yes	
116.	SNP (transistion)	189225	189227	A -> G				100%		No	No	Yes	
117.	SNP (transversion)	189252	189254	C -> G				100%		No	No	Yes	
118.	SNP (transversion)	189278	189280	T->A				14%	Pol16_20538_o9	No	No	No	
119.	Indel	189282	189283	Insertion T				100%		Yes	Yes	Yes	

Supplementary table 2. Characteristics of samples used in the study. Samples which gave positive results in virus isolation are shaded in grey, and titers obtained in 2nd passage are included in last column*.

Sample symbol	Number of Case (C) /Outbreak (O)	Origin (WB - wild boar/ DP - domestic pig)	Origin, tissue	Location	County	Collection date (D/M/Y)	*Titer in 2nd passage
P/14/16607	C12	WB	dead, spleen	Wiejki - Żubry	Białystok	30/07/2014	
P/14/18861	C14	WB	dead, spleen	Kolonia Mostowiany	Sokółka	24/08/2014	
P/15/32981	C81	WB	dead, bone marrow	Białowieża	Hajnówka	21/12/2015	
P/16/07631	C91	WB	dead, bone marrow	Budy	Hajnówka	15/04/2016	
P/16/15553	O4	DP	dead, spleen	Bielszczyzna	Hajnówka	23/06/2016	
P/16/15555		DP	dead, spleen	Bielszczyzna	Hajnówka	23/06/2016	
P/16/20186	O7	DP	dead, spleen	Izbiszcze Chorosz	Białystok	09/08/2016	6,74
P/16/20187		DP	dead, spleen	Izbiszcze Chorosz	Białystok	09/08/2016	7,32
P/16/20535	O9	DP	dead, spleen	Miodusy Stok	Wysokie Mazowieckie	10/08/2016	7,32
P/16/20538		DP	dead, spleen	Miodusy Stok	Wysokie Mazowieckie	10/08/2016	6,32
P/16/20540	O10	DP	dead, spleen	Truskolasy Niwisko	Wysokie Mazowieckie	10/08/2016	6,32
P/16/20778	O12	DP	dead, spleen	Augustowo	Bielsk Podlaski	11/08/2016	6,82
P/16/20804	O10	DP	dead, spleen	Truskolasy Niwisko	Wysokie Mazowieckie	12/08/2016	
P/16/20805		DP	dead, spleen	Truskolasy Niwisko	Wysokie Mazowieckie	12/08/2016	
P/16/20805		DP	dead, lymph node	Truskolasy Niwisko	Wysokie Mazowieckie	12/08/2016	
P/16/20806		DP	dead, spleen	Truskolasy Niwisko	Wysokie Mazowieckie	12/08/2016	
P/16/26002	O20	DP	dead, spleen	Nowe Szpaki	Łosice	08/09/2016	
P/16/29413	O23	DP	dead, spleen	Siemichocze	Siemiatycze	29/09/2016	7,52
P/16/43287	C156	WB	dead, spleen	Czeremcha	Hajnówka	13/12/2016	
P/16/43987	C160	WB	dead, spleen	Klukowicze	Siemiatycze	21/12/2016	
P/16/43988		WB	dead, bone marrow	Klukowicze	Siemiatycze	21/12/2017	
P/17/00016	C166	WB	dead, blood	Kolonia Dobryń Mały	Biała Podlaska	28/12/2016	

Sample symbol	Number of Case (C) /Outbreak (O)	Origin (WB - wild boar/ DP - domestic pig)	Origin, tissue	Location	County	Collection date (D/M/Y)	*Titer in 2nd passage
P/17/00117	C167	WB	hunted, spleen	Hoszczowa Królewska	Siemiatycze	01/01/2017	
P/17/02113	C195	WB	hunted, spleen	Hołowczyce Stare	Łosice	22/01/2017	
P/17/03028	C201	WB	dead, spleen	Nowe Litewniki	Łosice	30/01/2017	
P/17/03029		WB	dead, spleen	Nowe Litewniki	Łosice	30/01/2017	6,82
P/17/03030		WB	dead, spleen	Nowe Litewniki	Łosice	30/01/2017	7,32
P/17/04461	C210	WB	dead, spleen	Kolonia Litewniki	Łosice	11/02/2017	6,82
P/17/04463	C211	WB	hunted, spleen	Kolonia Litewniki	Łosice	14/02/2017	6,57
P/17/05838	C220	WB	dead, spleen	Niemirów	Siemiatycze	25/02/2017	7,32
P/17/05839		WB	dead, spleen	Niemirów	Siemiatycze	25/02/2017	
P/17/05840		WB	dead, spleen	Niemirów	Siemiatycze	25/02/2017	
P/17/05841		WB	dead, spleen	Niemirów	Siemiatycze	25/02/2017	
P/17/05842		WB	dead, spleen	Niemirów	Siemiatycze	25/02/2017	
P/17/05843		WB	dead, spleen	Niemirów	Siemiatycze	25/02/2017	
P/17/05951	C221	WB	dead, spleen	Niemirów	Siemiatycze	25/02/2017	
P/17/07333	C229	WB	dead, spleen	Nowa Kornica	Łosice	09/03/2017	
P/17/07668	C232	WB	dead, spleen	Borysowszczyzna	Siemiatycze	13/03/2017	
P/17/07852	C236	WB	hunted, spleen	Serpelice	Łosice	12/03/2017	
P/17/07852		WB	hunted, kidney	Serpelice	Łosice	12/03/2017	
P/17/07861	C235	WB	dead, spleen	Nowa Kornica	Łosice	11/03/2017	
P/17/07863	C234	WB	dead, spleen	Nowa Kornica	Łosice	14/03/2017	
P/17/08669	C245	WB	dead, spleen	Grabowiec	Siemiatycze	21/03/2017	
P/17/09017	C246	WB	dead, spleen	Sutno	Siemiatycze	22/03/2017	
P/17/10093	C249	WB	hunted, spleen	Stare Litewniki	Łosice	27/03/2017	
P/17/10093		WB	hunted, kidney	Stare Litewniki	Łosice	27/03/2017	
P/17/12669	C273	WB	hunted, spleen	Szpaki Kolonia	Łosice	17/04/2017	
P/17/12669		WB	hunted, kidney	Szpaki Kolonia	Łosice	17/04/2017	
